# Centrifugal Deposited Au-Pd Core-Shell Nanoparticle Film for Room-Temperature Optical Detection of Hydrogen Gas

**DOI:** 10.3390/s18051448

**Published:** 2018-05-06

**Authors:** Han Song, Zhijie Luo, Mingyao Liu, Gang Zhang, Wang Peng, Boyi Wang, Yong Zhu

**Affiliations:** 1 School of Mechanical and Electronic Engineering, Wuhan University of Technology, Wuhan 430070, China; lzjyingying@163.com (Z.L.); lmylyf@126.com (M.L.); 2 School of Mechanical Science and Engineering, Huazhong University of Science and Technology, Wuhan 430074, China; zhanggang@hust.edu.cn (G.Z.); pengwang@hust.edu.cn (W.P.); 3 School of Engineering, Griffith University, Nathan, QLD 4222, Australia; mr_williamw@hotmail.com (B.W.); y.zhu@griffith.edu.au (Y.Z.)

**Keywords:** optical fiber hydrogen sensor, Au-Pd core-shell nanoparticles, nanoparticles film, centrifugal deposition

## Abstract

In the present work, centrifugal deposited Au-Pd core-shell nanoparticle (NP) film was proposed for the room-temperature optical detection of hydrogen gas. The size dimension of 44, 48, 54, and 62 nm Au-Pd core-shell nanocubes with 40 nm Au core were synthesized following a solution-based seed-mediated growth method. Compared to a pure Pd NP, this core-shell structure with an inert Au core could decrease the H diffusion length in the Pd shell. Through a modified centrifugal deposition process, continues film samples with different core-shell NPs were deposited on 10 mm diameter quartz substrates. Under various hydrogen concentration conditions, the optical response properties of these samples were characterized by an intensity-based optical fiber bundle sensor. Experimental results show that the continues film that was composed of 62 nm Au-Pd core-shell NPs has achieved a stable and repeatable reflectance response with low zero drift in the range of 4 to 0.1% hydrogen after a stress relaxation mechanism at first few loading/unloading cycles. Because of the short H diffusion length due to the thinner Pd shell, the film sample composed of 44 nm Au-Pd NPs has achieved a dramatically decreased response/recovery time to 4 s/30 s. The experiments present the promising prospect of this simple method to fabricate optical hydrogen sensors with controllable high sensitivity and response rate at low cost.

## 1. Introduction

As one of the green energy carriers, hydrogen is one of the attractive measures for solving the energy supply security and the greenhouse gas reduction because of its favorable energy density, renewability, and eco-friendly nature. However, the gaseous hydrogen is highly flammable and explosive in a wide volume range (4–75%) in the air condition [1]. As an essential device for the concentration detection and leakage monitoring of the hydrogen gas, hydrogen sensors are required to have rapid response rate, high sensitivity, and reliability to provide early warning before the concentration reaches the low explosion limit 4%. They are large in demand to ensure safety in the production, storage, delivery, and fueling processes of hydrogen energy.

Superior to conventional electrochemical sensors, the optical fiber hydrogen sensor shows applicable value to the hazardous or smeary industrial environment because of its intrinsic safety, corrosion resistance, and anti-electromagnetic interference. Palladium (Pd) and its alloy or composite films are mostly utilized as sensitive components in optical fiber hydrogen sensors for the high sensitivity and selectivity of Pd to the hydrogen gas. Due to changes of the mechanical stress and optical constants triggered by the hydrogenation process of Pd films, optical hydrogen sensors based on various modulation methods have been reported, such as signal intensity [2], wavelength [3], interference path length [4], and polarization state [5]. In this case, the process of Pd-hydride formation plays a crucial role in the response behavior of these sensors. The response speed of the sensors is determined by not only the gas molecule adsorption, dissociation (H_2_→2H), and H atoms diffusion processes in Pd-based films, but also an *α*→*β* phase transition reaction of the Pd-hydride formation at the high level of hydrogen [6].

Perrotton et al. [7] proposed a composite film with 2.5 nm ultra-thin Pd layer coated on a sensitive fiber surface plasma resonance (SPR) structure for optical hydrogen detection. Benefit from the short H diffusion length of the Pd nanolayer, the sensor exhibited a fast response and recovery time as 3 s and 10 s for 4% hydrogen. Zhao et al. [6] investigated several Pd alloys, including Pd0.94-Ag0.06, Pd0.94-Au0.06, and Pd0.6-Au0.4 as all-optical hydrogen-sensing materials. It was demonstrated that the alloy film with high Au doping (40% in mass ratio) could effectively inhibit the phase transition reaction to achieve a higher response speed. After that, Monzón-Hernández et al. [8,9] presented direct-deposited Pd-Au 20 nm film and annealed Pd-Au bilayer film stack (Pd 1.4 nm and Au 0.6 nm with 1~5 stacks) for an evanescent-field hydrogen sensor. The 2 stacks film with 4 nm thickness showed a rapid response and recovery time of 4.5 s and 13 s for 4% hydrogen. However, the response value of this sensor was small and decline sharply from 4 to 2% hydrogen, which makes the signal changes of individual stack difficult to measure. In our previous work [10,11], Pd-Y alloy film was proposed to suppress the phase transition reaction to fabricate an optical hydrogen sensor with fast response. 10-nm thin Pd-Y sensitive film was demonstrated a response/recovery rate at 6 s and 8 s to 4% hydrogen on a reflective fiber-bundle sensor. Through an annealing-stimulated process, it achieved a sensitivity and stability enhancement with less than 5% zero drift in multiple cycles from 4 to 0.1% hydrogen range.

Nanoscale sensitive materials are attractive to improve the hydrogen sensing performance because the high surface-to-volume ratio increases the contact area to the gas molecule and correspondingly decreases the diffusion length. Moreover, the miscibility gap between *α* and *β* phase of the Pd-H system, as well as the critical threshold temperature of phase transition decrease with the particle size decreases [12]. It presents a direct method to suppress phase transition reaction through the size effects of the nanoscale materials themselves. In studies of electronic hydrogen sensors, Pd nanostructures, including nanorods [13], nanowires [14], and NPs [15] shown a promising prospect not only for their high response rate and sensitivity, but also for their small size, flexibility, and capability to realize multi-sensor integration. For optical hydrogen detection, Sirbuly et al. [16] proposed a high porosity cladding stabilized Pd NPs to modulate the intensity of evanescent-field under 3 s in a hydrogen concentration range from 0.8% to 8%. Monzón-Hernández et al. [17] utilized an annealing process to transform 2 nm Pd-Au bilayer film, which sputtered on a fiber taper device into disconnected NPs and obtained 2 s in response to 4% hydrogen. However, the microscopic images exhibit a few and scattered particles adhering to the above two kinds of evanescent field sensing devices, leading to a low response amplitude in hydrogen measurement. Wadell et al. [18] proposed arrays of nanofabricated Pd-Au alloy NPs as plasmonic optical hydrogen sensors. The Pd-Au alloy NPs could suppress the hysteresis and increase the sensor accuracy and sensitivity. An improved sensor response time to below one second achieved within the 0−40 mbar pressure range by utilizing 25 atom % Au alloy NPs. Other Pd nanostructures, such as single Pd-Au nanofocus [19] and single Pd nanowire [20], have been studied for their high sensitivity, but the weak modulation effects of the single nanostructure request expensive apparatus. Also, the size dimension of the Pd nanoplate and nanowire nearly 100 nm diameter limits their response rate.

In the present work, we focus on the optical response performance of hydrogen sensitive films composed of Au-Pd core-shell NPs. Compared to a pure Pd NP, this core-shell structure with an inert Au core could decrease the H diffusion length in the Pd shell. Moreover, the response properties could be adjusted by controlling the thickness of the shell. Following a solution-based seed-mediated growth method, the Au-Pd core-shell NPs with various thickness of Pd shell were synthesized. Then, deposited on a quartz substrate, respectively, by using a modified centrifugal deposition method. UV-vis spectrophotometer was used to characterize the absorption spectrum of the nanocrystal solution. XRD, TEM, and SEM were utilized to determine the morphology of both the sensitive NPs and the films. Through a proposed reflective optical fiber bundle sensor system, the hydrogen response of these Au-Pd core-shell nanocrystal film samples was measured. This method is independent of precision equipment and apparatus (such as sputter coater, rotary evaporator, or atomic layer), and is easy to realize the light path system. It has presented a simple and promising means to fabricate a hydrogen optical fiber sensor with high performance and low cost.

## 2. Materials and Methods

### 2.1. Materials

Gold chloride trihydrate (HAuCl_4_·3H_2_O, ≥99.9%), Palladium chloride (PdCl_2_, 59–60% Pd), ascorbic acid (AA, ≥99%), sodium borohydride (NaBH_4_, 98%), cetyltrimethylammonium bromide (CTAB, 99%), and ethanol (≥99.5%) were purchased from Aladdin (Shanghai, China). HCl (20 mM standard solution) was acquired from Kemiou (Tianjin, China). All chemicals were used as received. Deionized water (18 MΩ cm^−1^) was obtained from an ultra-pure water purifier (Milli-Q). Polypropylene (PP) substrates with 30 mm diameter were used to collect enough amounts of NPs for the XRD characterization. Moreover, quartz glass substrates with 10 mm diameter were used for sensitive films deposition for the SEM characterization and hydrogen response test.

### 2.2. Preparation of Au NPs

The Au NPs suspension was synthesized in aqueous solution following a seed-mediated growth method reported by TK Sau et al. [21]. First, a 10 mL solution of Au seeds was prepared by the reduction of HAuCl_4_ (2.5 × 10^−4^ M) with ice-cold NaBH_4_ (6.0 × 10^−4^ M) in the presence of CTAB (7.5 × 10^−2^ M) at room temperature. The mixture was magnetically stirred for two min until the solution turned orange, indicating the formation of Au seeds. 

In a typical growth reaction, a 200 mL solution containing HAuCl_4_·3H_2_O (2.5 × 10^−4^ M), AA (6.0 × 10^−3^ M), and CTAB (1.6 × 10^−2^ M) was prepared as a growth solution. Then, a 10 μL solution of freshly prepared Au seeds was added into the growth solution with stirring until the reaction mixture quickly turned the initial orange solution into pink. Finally, the solution was left undisturbed for 15 min.

### 2.3. Preparation of Au-Pd Core-Shell NPs

For the synthesis of Au-Pd core-shell NPs, a 25 mL solution of H_2_PdCl_4_ (10 mM) was prepared by completely dissolving 44.5 mg PdCl_2_ in 25 mL standard HCl solution (20 mM). Then, 25, 50, 100, and 200 μL of 10 mM H_2_PdCl_4_ solution were mixed into 10 mL Au NPs solution, respectively, and kept at 50 °C for 2 h. Result from the reducing reaction between the Pd^2+^ and the excessive amount of AA in the Au NPs solution, 44, 48, 54, and 62 nm Au-Pd core-shell NPs corresponding to the introduced 25, 50, 100, and 200 μL growth solution were synthesized with a same 40 nm cubic Au core. The shape and size of all the NPs samples were determined in the later characterizations. The freshly prepared Au-Pd core-shell NPs solution samples were centrifuged at 4000 rpm for 10 min to remove the residual CTAB surfactant and redispersed in the 10 mL of deionized water.

### 2.4. Centrifugal Deposition of NPs Film

The prepared Au-Pd core-shell NPs were deposited on quartz glass substrates by using a modified centrifuge sedimentation method reported by Markelonis et al. [22]. The horizontal rotor centrifuge was manufactured by Xiangyi Centrifuge Instrument Corporation (Cence, TD5A-WL, Changsha, China). In this study, the 10 mm diameter quartz glass substrate with both sides polished was placed in the bottom of a 12 mm diameter vial. Then, 0.5, 1, 2, and 4 mL of prepared Au-Pd core-shell NPs solutions were diluted to 5 mL by 30% (volume concentration) ethanol solution. The samples were transferred to the vials, respectively, and then sonicated for 2 min to make sure the NPs were dispersed well in the solutions. Finally, the vials were placed in the horizontal rotor centrifuge and spun for 8 min at 4000 rpm. After removing the supernatant, the substrates with various thickness of Au-Pd core-shell NPs deposition were took out and dried in nitrogen for subsequence tests. 

### 2.5. Hydrogen Sensing Experimental Setup

**Figure 1:**
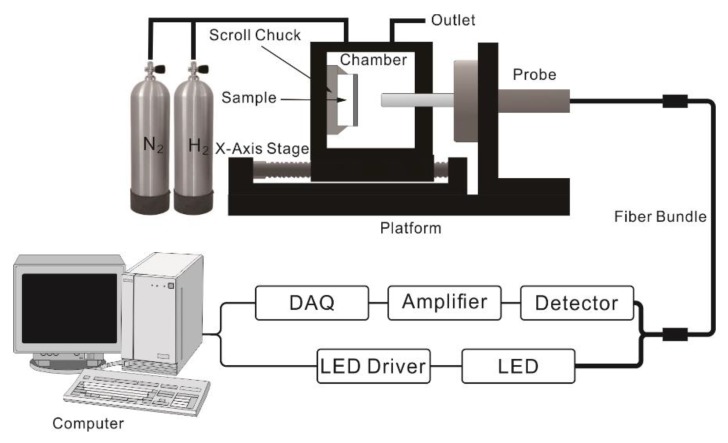
Schematic of reflective optical fiber bundle sensor.

In this work, a reflective optical fiber bundle sensor has been realized to characterize the hydrogen response performance of Au-Pd core-shell NP film samples. As shown in Figure 1, a high-power LED source (FCS-0625, Mightex Systems, Toronto, ON, Canada), which central wavelength is 625 nm is coupled into a Y-type fiber bundle to provide the computer-controlled light pulse. The light signal is projected onto the sample in a gas chamber through the fiber bundle. Then, the reflective signal returning from the sample is collected by a silicon photodiode detector (PDA8A, Thorlabs, Newton, NJ, USA) at another end of this fiber bundle and transfer to voltage signal. Result from the volume density reduction of free electrons after hydrogen exposure, both the real and imaginary parts of the complex permittivity of Pd decrease [23]. The intensity of the output signal is determined by the complex refractive index of the NPs film.

## 3. Results and Discussion

### 3.1. Characterization NPs

**Figure 2:**
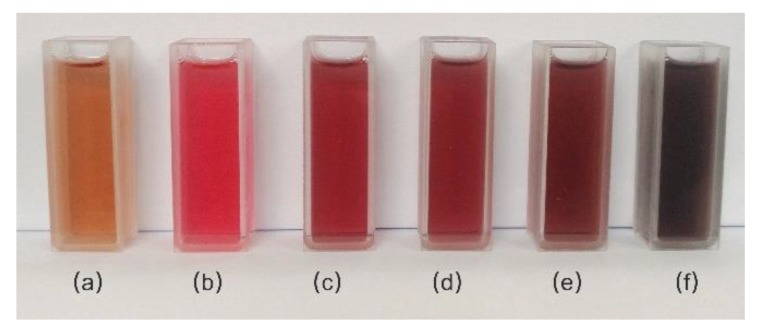
Photograph of synthesized NPs solution in 10 mm path-length cuvette (**a**) Au seeds; (**b**) Au NPs; (**c**–**f**) 44 nm, 48 nm, 54 nm, and 62 nm Au-Pd core-shell NPs, respectively.

The photograph of synthesized NPs solution samples is shown in Figure 2. All the prepared samples have the same particle concentration, which is determined by the amount of initial introduced Au seeds solution. Through a crystal growth process from Au seeds, the particle size of the Au NPs increases to around 44 nm with a color change from orange to pink. The thickness of Pd shell is according to the reduction amount of the H_2_PdCl_4_. Due to the addition of a various amount of Pd growth solutions, the absorbance of the Au-Pd core-shell NPs samples increases with an increase in the thickness of Pd shell, leading to color changes from pink to crimson, carmine, wine red and dark red, respectively.

**Figure 3:**
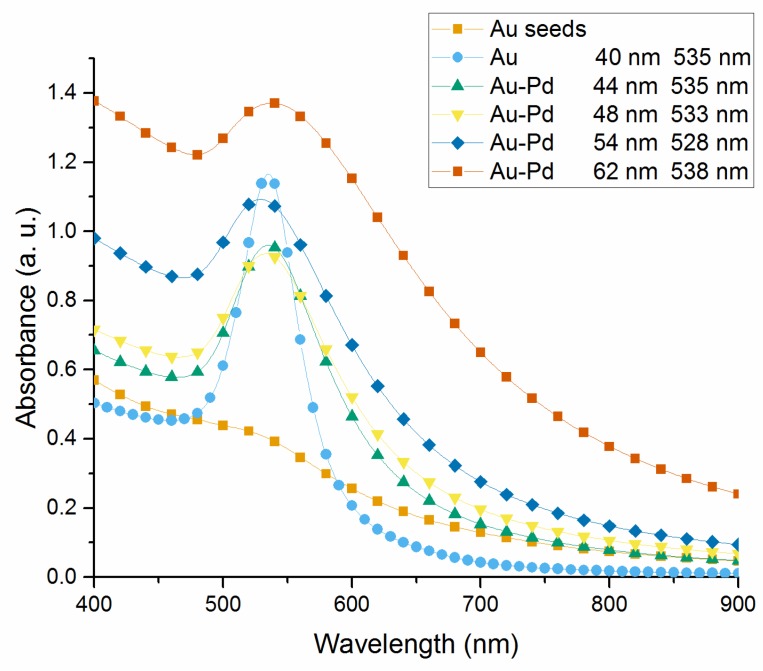
Absorption spectra of the synthesized NPs UV-vis absorption spectra of Au seeds, 40 nm Au NPs, 44, 48, 54, and 62 nm Au-Pd core-shell NPs in deionized water; the corresponding absorption peak of each sample is listed in the legend.

The absorbance spectrum of the Au and Au-Pd core-shell NPs colloidal solutions are measured by using an UV-visible light spectrophotometer (Lambda 35, Perkin-Elmer, Norwalk, CT, USA) with deionized water as a reference. The absorption spectra of the Au seeds, 40 nm Au NPs, 44, 48, 54, and 62 nm Au-Pd core-shell NPs solutions are shown in Figure 3. The spectrum of Au seeds solution shows no obvious SPR band at range 300 nm to 900 nm, while the Au core grown to 40 nm shows a single SPR band at 533 nm. With an increased Pd layer thickness, the absorbance exhibits an upward trend. In addition, the SPR band in the absorption spectrum shows a broadened width and weakened intensity. An initial blue-shift of the SPR peak occurs from 533 nm to 528 nm, corresponding to the Pd layer thickness grows from 2 nm to 7 nm, but it is followed by a red-shift to 538 nm with further growth of Pd shell to 11 nm.

**Figure 4:**
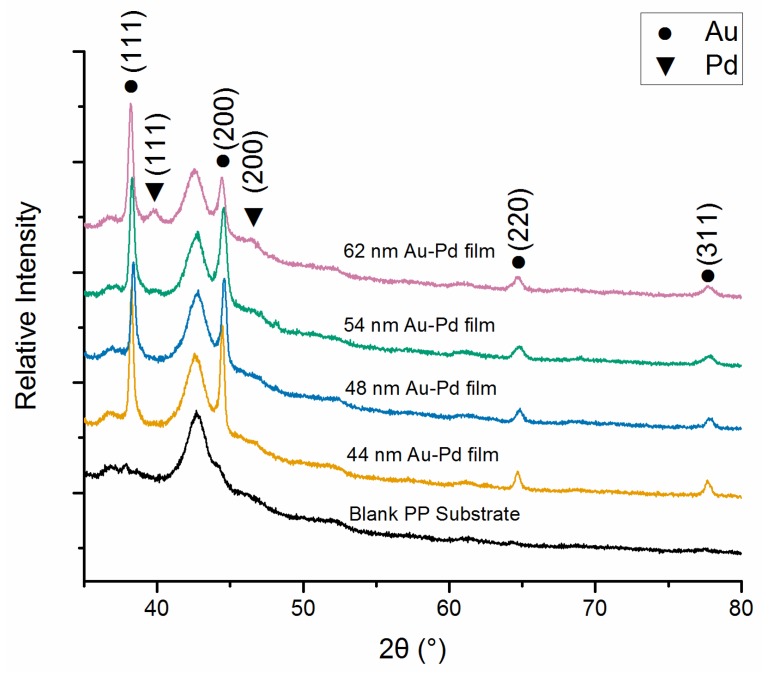
XRD patterns of 44 nm, 48 nm, 54 nm and 62 nm Au-Pd core-shell NPs films and the blank PP substrates.

By using the same centrifuged deposition method, the Au-Pd core-shell NPs were deposited on PP substrate for XRD test. The XRD patterns of these samples are collected and compared with the standard powder diffraction card for Au (No. 04-0784) and Pd (No. 46-1043), as shown in Figure 4. It shows that the (111), (200), (220), and (311) planes of Au are obviously detected in the XRD spectrum. The (111) peak intensity for Au is much stronger than the other ones. Moreover, the reflection peaks of Au are relatively stronger than those peaks for Pd due to the relatively thin Pd shell thickness. Corresponding to the increased molar ratio of Pd: Au (1:10, 1:5, 2:5, and 4:5 for 44 nm, 48 nm, 54 nm, and 62 nm Au-Pd NPs, respectively), the (111) and (200) peaks of Pd gradually appeared. Intensities of the (111) peak for Pd are slightly higher than the (200) peak, implying a similar orientation on the substrate surface as Au. The theta angle of Pd (111) and (200) peaks are 39.92 and 46.38, respectively, which have shifted to lower by about 0.19 and 0.27, with respect to the standard peak positions. As compared to Pd, the shift of Au reflection peaks is hardly observed. It indicates that the Pd shell has a lattice expansion in its growth process, which is mainly attributed to the different atomic radii of Au and Pd (around 4.6%) induced strain at the core-shell interface [24].

**Figure 5:**
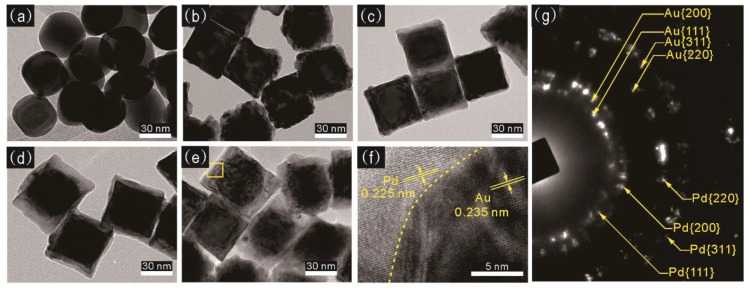
(**a**) TEM image of 40 nm Au core; (**b**–**e**) TEM images of the Au-Pd core-shell NPs with different size of 44, 48, 54, and 62 nm after the addition of a various amount of H_2_PdCl_4_ solution; (**f**) HR-TEM image of the square regions shown in panel (**e**); (**g**) the selected-area electron diffraction (SAED) patterns of panel (**e**).

The synthesized NPs samples were further characterized by TEM. In Figure 5a, it is determined that the Au core nanocrystal has a round cube shape with a dimension size of 40 nm. Through a reduction of the various amount of H_2_PdCl_4_ solution, the Pd shell formed around the Au core that leads to an increased dimension size to 44, 48, 54, and 62 nm, with a more squared particle shape, which is shown in Figure 5b–e. Figure 5f is a representative HR-TEM image that focuses on the crystalline structures of the 62 nm core-shell NPs. It shows that a clear interface could be observed between the Au core and Pd shell. The fringes have a spacing of ∼0.225 nm and ∼0.235 nm for the (111) planes of Pd and Au, respectively. The (111) planes of Pd aligns parallel to the (111) planes of Au, presenting that Pd grows epitaxially on the {111} faces of the Au NPs from the nanocube corner. Figure 5g shows selected-area electron diffraction (SAED) pattern of 62 nm Au-Pd core-shell NPs. The concentric rings shown in the SAED pattern indicate that the NPs have a crystalline state. The diffraction rings could be assigned to the Au and Pd fcc phase correlate with (111), (200), (220), and (311) diffractions, respectively.

### 3.2. NPs Film Characterization

**Figure 6:**
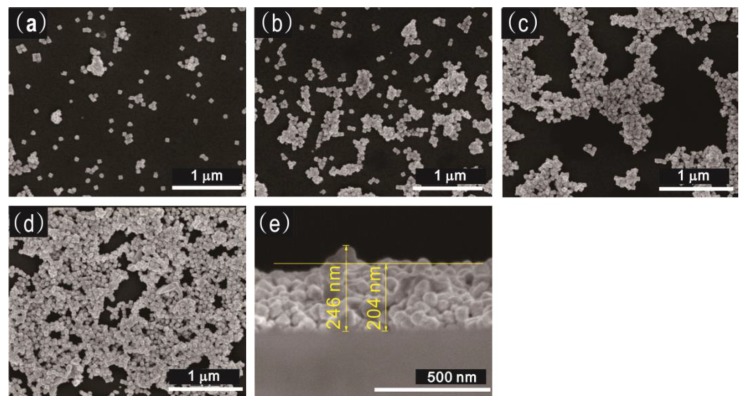
(**a**–**d**) SEM images of the centrifugal deposited 62 nm Au-Pd core-shell NPs film with 0.5, 1, 2, and 4 mL solution; (**e**) cross-section image of the sample in panel (**d**).

The SEM images of the surface morphology of deposited 62 nm Au-Pd core-shell NPs film samples are given in Figure 6. The NP films exhibit an aggregation trend from isolated NP in panel (a), partial coalescence in panel (b), coterminous NPs in panel (c) to continues film in panel (d), which is corresponding to the amount of the introduced NPs solution increased from 0.5 to 4 mL. The coverage of these four samples is 8.5%, 22.2%, 39.5%, and 78.8%, respectively, which is roughly parallel to the volume ratio of NPs solutions as 1:2:4:8. The cross-section of the continues film shown in panel (d) is shown in Figure 6e, the average thickness of this sample is around 204 nm and the max thickness is 246 nm.

### 3.3. Hydrogen Response Test

**Figure 7:**
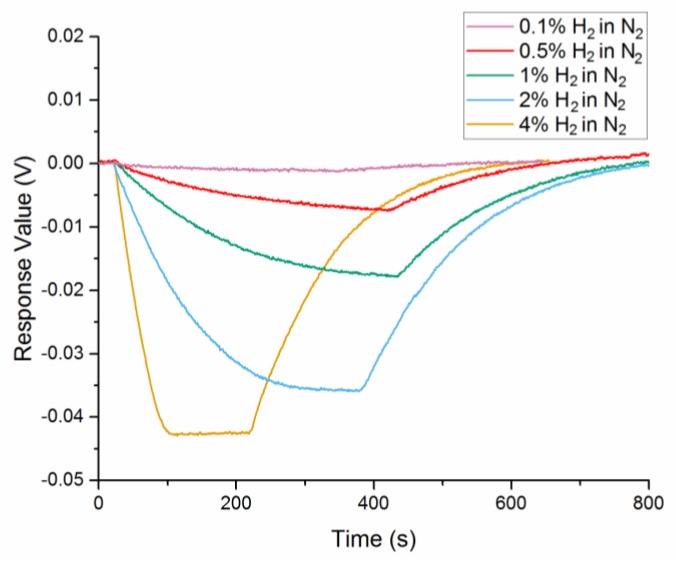
Response curve of 62 nm Au-Pd core-shell NPs film in 2%, 1%, 0.5%, 0.1% hydrogen.

The response curve of 62 nm Au-Pd core-shell NPs film in 4%, 2%, 1%, 0.5%, 0.1% hydrogen at room temperature and pressure is presented in Figure 7. The response time, recovery time, and response amplitude of each curve are listed in Table 1. The response and recovery times are defined as the time when 90% of the full signal change induced by hydrogen absorption/desorption is achieved. 

As the concentration of hydrogen decreased from 4 to 0.1%, the response time raised from 62 s to 330 s. However, the recovery time shows a minor fluctuation, which gets a maximum of 401 s in 2% hydrogen and a minimum of 318 s in 0.1% hydrogen.

**Figure 8:**
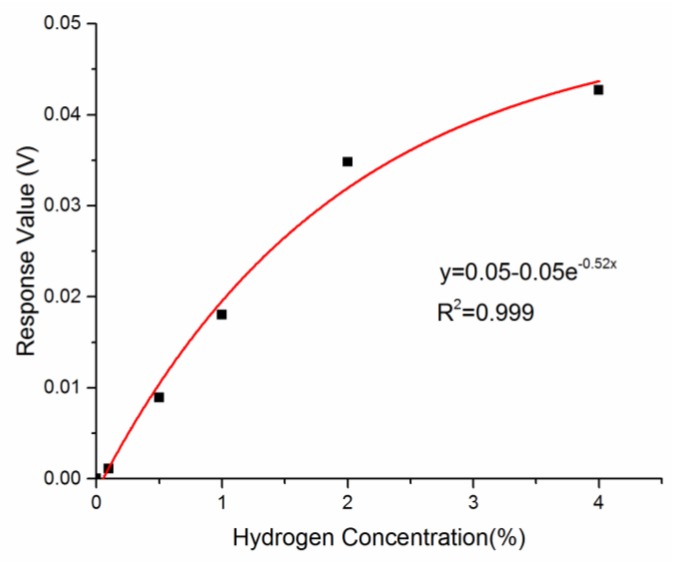
Curve fitting response value of hydrogen sensor under different concentration (4%, 2%, 1%, 0.5%, and 0.1% Hydrogen).

Figure 8 plots the curve fitting of response amplitude of the sensor under various hydrogen concentration. A nonlinear fitting (exponential function) with a standard error of R^2^ = 0.999 shows an increased sensitivity, corresponding to the hydrogen concentration (2.54, 2.30, 2.03, 1.62, 1.10 mV/% for 0.1%, 0.5%, 1%, 2%, 4% hydrogen, respectively).

**Figure 9:**
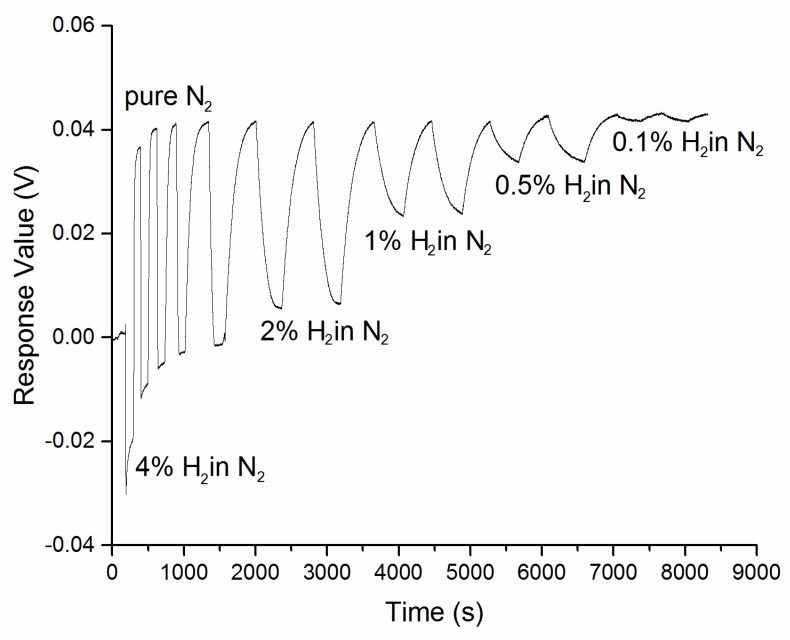
Multi-cycle measurement of 62 nm Au-Pd core-shell NPs film exposing to different concentration of hydrogen (4%, 2%, 1%, 0.5%, and 0.1% in nitrogen).

As shown in Figure 9, the multi-cycle reflective response of 62 nm Au-Pd core-shell NPs film to 0.1~4% hydrogen (using nitrogen as both carrier and recovery gas) has been measured.

After a 38 mV zero drift in the first absorption/desorption cycle, the reflective response of the film tended to a stable zero point and shown high repeatability at 43 mV. This phenomenon may be attributed to a relaxation mechanism of the residual stress within the particles film during the centrifugal deposition, which is similar to our previous study of sputtering Pd films [11]. Even though a 5 mV zero drift occurred in the next three cycles, the response amplitude maintained at 43 mV with an error less than 5%.

**Figure 10:**
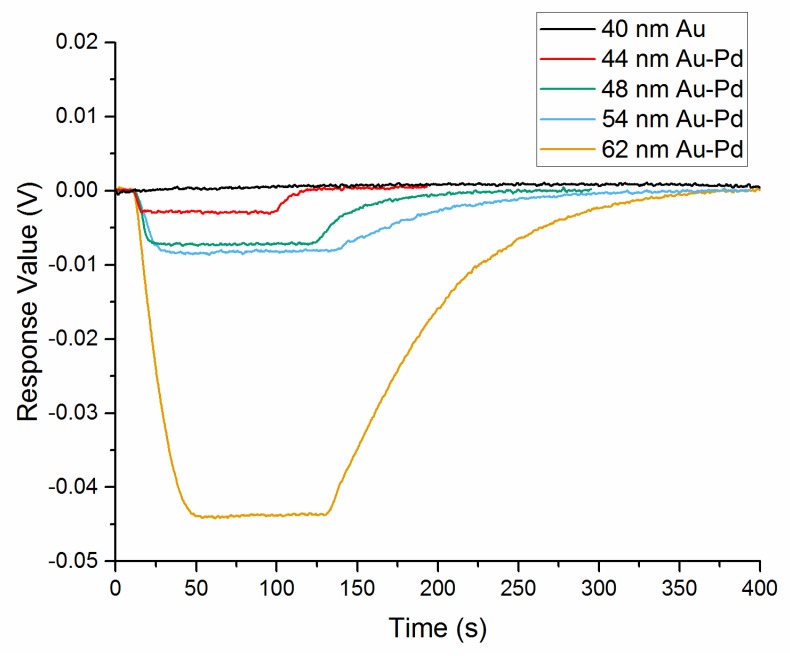
Response curve of 40 nm Au core and 44 nm, 48 nm, 54 nm, 62 nm Au-Pd core-shell nanocrystals films to 4% hydrogen in nitrogen.

The 4% hydrogen response curve for 40 nm Au core and 44 nm, 48 nm, 54 nm, 62 nm Au-Pd core-shell NPs films after a few of curing cycles has been presented in Figure 10. For 40 nm Au NPs film, the sensor exhibited a fluctuation in order of 0.1 mV. It demonstrated that the reflectance changes just depended on the hydrogenation and dehydrogenation of the Pd layer. The response parameters to each kind of NPs film are listed in Table 2. From the experimental results, there is a complete positive correlation between the decrease in thickness of Pd shell and the reduction in response/recovery time of the sensor. The response amplitude declines sharply from 43.9 mV for the 62 nm size Au-Pd NPs film to 2.8 mV for the 44 nm size sample. As a result of the decreased diffusion length of Pd layer, the response/recovery time reduced to 4s/30s for 44 nm size sample compared to 62 s/348 s for 62 nm size sample. It is promising for such sensor to be employed in fast hydrogen detection in low concentration range under 4%.

The compared performances of hydrogen sensors in reference are shown in Table 3. The sensor based on Au-Pd core-shell NPs film prepared in this paper has a wide measurement range as 0.1–4%. Without a further thermal treatment, the film composed of 44 nm Au-Pd NPs has achieved improved response speed than the alloy film in Reference [6], as 4 s response to 4% hydrogen.

## 4. Conclusions

In summary, the hydrogen response characteristics of centrifugal deposited Au-Pd core-shell NPs films have been determined by an intensity-based optical fiber bundle sensor at room temperature. The Au-Pd core-shell NPs with various particle sizes were synthesized by a seed-mediated growth method in aqueous solution. Through a 4000 rpm centrifugal deposition, a 204 nm sensitive film aggregated from 4 mL NPs solution formed on 10 mm diameter quartz substrate. The experimental results show that the NPs film composed of 62 nm Au-Pd NPs achieves a nonlinear increased sensitivity to hydrogen concentration ranging from 4 to 0.1%. After a stress relaxation mechanism at the first loading/unloading cycle, this film exhibits a stable and repeatable reflectance response with low zero drift in the measurement of various hydrogen concentration. By controlling the thickness of Pd layer, it has demonstrated a complete positive correlation between the decrease in thickness of Pd shell and the reduction in response/recovery time of the sensor. A fast response/recovery time as 4 s/30 s to 4% hydrogen is achieved for 44 nm Au-Pd core-shell NPs film. The experiments present the promising prospect of this simple method to fabricate optical hydrogen sensors with controllable high response performance at low cost. These synthesized Au-Pd core-shell NPs are applicable for scale production of sensitive components for other kinds of hydrogen sensors.

## Figures and Tables

**Table 1:** Response parameters of the 62 nm Au-Pd core-shell NPs film to different concentration of hydrogen (2%, 1%, 0.5%, and 0.1% in nitrogen).

Hydrogen Concentration (%)	Response Amplitude (mV)	Response Time (s)	Recovery Time (s)
4%	43.9	62	348
2%	34.8	190	401
1%	18.0	270	349
0.5%	8.9	300	363
0.1%	1.2	330	318

**Table 2:** Response parameters of 40 nm Au core and 44 nm, 48 nm, 54 nm, 62 nm Au-Pd core-shell NPs films to 4% hydrogen in nitrogen.

Film Component	Response Amplitude (mV)	Response Time (s)	Recovery Time (s)
40 nm Au	0	-	-
44 nm Au-Pd	2.8	4	30
48 nm Au-Pd	7.1	8	94
54 nm Au-Pd	8.4	14	159
64 nm Au-Pd	43.9	62	348

**Table 3:** Compared performances of hydrogen sensors in reference.

Author, Reference	Sensing Film Component	Concentration Range	Response Time
This paper	44 nm Au-Pd core-shell NPs film	0.1–4%	4 s at 4% H_2_
Zhao [6]	20 nm Pd_0.6_Au_0.4_ alloy film	0.05–4%	5 s at 4% H_2_
Perrotton [7]	2.5 nm Pd/35 nm Au/180 nm SiO_2_ composite film	0.5–4%	3 s at 4% H_2_
Sirbuly [16]	POSS-Pd NPs	0.8–8%	3 s at 3% H_2_
Monzón-Hernández [17]	Annealed Au-Pd NPs	0.8–8%	2 s at 4% H_2_
Wadell [18]	Annealed Au-Pd alloy nanodisk	Up to 4%	<1 s at 4% H_2_
